# Feeling safe or unsafe in psychiatric inpatient care, a hospital-based qualitative interview study with inpatients in Sweden

**DOI:** 10.1186/s13033-019-0282-y

**Published:** 2019-04-08

**Authors:** Veikko Pelto-Piri, Tuula Wallsten, Ulrika Hylén, Iradj Nikban, Lars Kjellin

**Affiliations:** 1 University Health Care Research Center, Faculty of Medicine and Health, Örebro University, Örebro, Sweden; 2 Centre for Clinical Research, Uppsala University, County Hospital Västerås, Västerås, Sweden; 3 Nikban Psykoterapi AB, Stockholm, Sweden

**Keywords:** Psychiatry, Inpatients, Safety, Violence, Values, Qualitative interviews, Thematic content analysis

## Abstract

**Background:**

A major challenge in psychiatric inpatient care is to create an environment that promotes patient recovery, patient safety and good working environment for staff. Since guidelines and programs addressing this issue stress the importance of primary prevention in creating safe environments, more insight is needed regarding patient perceptions of feeling safe. The aim of this study is to enhance our understanding of feelings of being safe or unsafe in psychiatric inpatient care.

**Methods:**

In this qualitative study, interviews with open-ended questions were conducted with 17 adult patients, five women and 12 men, from four settings: one general psychiatric, one psychiatric addiction and two forensic psychiatric clinics. The main question in the interview guide concerned patients’ feelings of being safe or unsafe. Thematic content analysis with an inductive approach was used to generate codes and, thereafter, themes and subthemes.

**Results:**

The main results can be summarized in three themes: (1) *Predictable and supportive services are necessary for feeling safe.* This concerns the ability of psychiatric and social services to meet the needs of patients. Descriptions of delayed care and unpredictable processes were common. The structured environment was mostly perceived as positive. (2) *Communication and taking responsibility enhance safety.* This is about daily life in the ward, which was often perceived as being socially poor and boring with non-communicative staff. Participants emphasized that patients have to take responsibility for their actions and for co-patients. (3) *Powerlessness and unpleasant encounters undermine safety.* This addresses the participants’ way of doing risk analyses and handling unpleasant or aggressive patients or staff members. The usual way to act in risk situations was to keep away.

**Conclusions:**

Our results indicate that creating reliable treatment and care processes, a stimulating social climate in wards, and better staff-patient communication could enhance patient perceptions of feeling safe. It seems to be important that staff provide patients with general information about the safety situation at the ward, without violating individual patients right to confidentiality, and to have an ongoing process that aims to create organizational values promoting safe environments for patients and staff.

**Electronic supplementary material:**

The online version of this article (10.1186/s13033-019-0282-y) contains supplementary material, which is available to authorized users.

## Background

A major challenge in psychiatric inpatient care is to create an open and rehabilitative environment that promotes patient recovery, patient safety and a good working environment for staff. Staff members need to take positive risks in their work with patients by gradually bringing back responsibility and initiative to the patient [1, 2]. At the same time, violence in the ward may negatively affect patient recovery [3, 4], staff health [5, 6], and the organization [7]. Therefore, it is important to create a safe environment through primary preventive interventions so that both staff and patients can feel safe.

Most health care in Sweden is publicly funded and run by regional councils. Over the past few decades, Sweden, like many other Western societies, has the latest decades invested in outpatient care and made a radical reduction in the number of beds in psychiatric inpatient care. As a result, the proportion of inpatients receiving coercive care due to serious psychiatric conditions has increased [8, 9]. In psychiatric inpatient care in Sweden, 83% of nursing staff have reported experiences of violence; 47% in the previous 6 months [10]. It is, therefore, necessary to minimize violence, including self-harm and coercive measures, in order to create a safe environment. Research on the prevention of violence implies that management and staff ability to create a good ward environment has a crucial influence on the risk of violence [11]. The wide variation in the use of coercive measures can not only be explained by patient diagnoses or other patient variables [12]. Instead, it appears that some institutions are more successful than others in creating a safe environment that helps to minimise the frequency of coercive measures [13, 14]. Situations where staff members need to restrict patient freedom or deny patients their wishes have been found to explain 39% of violence from patients in psychiatric inpatient care in Europe [15]. The Safewards Model includes six domains that can be used by management and staff as a basis for modifying risk factors: the physical environment, the staff team, the patient community, patient characteristics, outside hospital and the regulatory framework, [11, 13]. The program is widely used and available in seven languages [16]. On an organizational level, the management can give prerequisites for the prevention of violence through good managerial policies, organizational values and an efficient organization with a clear purpose of care [13, 14]. These organizational factors affect the ward structure and make it easier for ward managers and staff to create consistent and reasonable ward rules. It is essential that staff learn to communicate with patients effectively and in a caring manner as well as endeavouring to understand the reason or trigger for a patient’s aggression. Other preventive measures are to take care of agitated patients at an early stage and to use de-escalation methods when appropriate [13, 17, 18].

In this study, the focus is on the patients and the factors that make them feel safe or unsafe in psychiatric inpatient care. Being safe as a patient is not only about physical safety but also about the broader context of the general atmosphere of feeling safe in the ward. Many features in guidelines and programs to manage violence are in line with the recovery approach such as involving patients in all decisions about their care and treatment and improving their experience of staying in the ward [19] forming supportive relationships, giving hope, meaningful activities and developing coping skills [13, 20]. This implies that work with safety and quality of care needs to be integrated in order to be beneficial to patients in mental health care [21]. In studies of patient views on psychiatric inpatient wards, patients have reported that they appreciate staff who communicate and create a therapeutic relationship with them. Such staff can promote a sense of trust and safety [22–27], help to reduce patient anxiety [26] and resolve conflicts [27, 28]. Good communication with patients can result in patients feeling valued and more human [26, 29, 30]. On the other hand, factors such as staff not being seen enough by the patients, lack of communication and staff not showing understanding of the patient’s illness, as well as stigmatizing remarks have resulted in patients feeling that they are not being respected and are less valuable than other humans [22, 31, 32], which can lead to violent behaviour [27]. Rules of the ward can create conflicts if they are difficult to understand, are rigid and their application is perceived as arbitrary [26, 32, 33]. Patients may perceive that they have to adapt to the ward environment, have days without meaningful activities and accept changes in medication without being consulted [22, 27, 32, 34–36]. They may perceive that if they do not adapt, or show negative emotions, they might be subjected to coercive measures or be frightened by staff into adapting [22, 31–33]. Patients can notice that staff are not always sensitive in detecting patients whom they consider being a safety risk [37]. Sometimes they feel they are being stalked by another patient [38, 39], have problems with co-patients using alcohol or drugs or are victims of theft of personal possessions at the ward [38]. In these situations, it is important to have an own room to go to, as a lack of personal space has been described as problematic [27, 32, 38, 39]. Patients reported feeling safe from others outside the clinic and at lower risk for self-harm, in addition, male staff gave a higher sense of physical protection than female staff [39].

Guidelines and programs stress the importance of primary prevention in creating safe environments. Most of the studies referred to above aimed to describe how patients perceive psychiatric inpatient care in general. Since only a few of the studies have focused on patients own perceptions of feeling safe, there is need for further research in order to understand what we should improve to achieve effective primary prevention. When we interviewed staff, they emphasized that creating a relationship and good communication were prerequisites both for good care and for primary violence prevention [40]. In the present study we wanted to interview patients in these wards and ask them how they perceive safety issues. The aim of this study is to enhance our understanding of feelings of being safe or unsafe in psychiatric inpatient care from a patient perspective in the ward environment by letting patients freely express their views on safety issues.

## Methods

### Participants and settings

We interviewed 17 patients treated in four inpatient settings for adults in three Swedish regions; one general psychiatric, one psychiatric addiction and two forensic psychiatric clinics; one with low, and the other with medium, security class. The first three clinics mentioned had patients in need of psychiatric in-patient care from the respective surrounding catchment areas. Patients in all of these clinics were cared for because they had a psychiatric diagnosis. Substance users were found in all clinics, but the psychiatric addiction clinic also had special competence to care for patients with co-occurring addiction and psychiatric problems. The medium security clinic had a high perimeter security in comparison to the low security clinic. None of them had staff with police authority. The medium security clinic had patients from surrounding catchment areas and also from other areas of Sweden. During the time of the study, all clinics intended to provide single rooms for all patients. We used purposive sampling and chose these settings to get a wide range of clinics. For this study, patients were recruited by the ward managers in the psychiatric clinics who gave them verbal and written information. If the patient agreed to participate, we were informed. We wanted to get as wide a range as possible regarding age and gender. Managers asked patients whom they deemed able to participate, who had begun to recover and had been patients for so long that they had experiences to share. Patients also received written information about the study. Five of the participants were women and 12 were men; their ages ranged from their twenties up to 67 years.

### Design and procedure

This is an interview study focusing on the question of feeling safe or unsafe, with some optional open-ended questions. Thematic content analysis with an inductive approach was used to generate codes and thereafter themes and subthemes.

An interview guide was created after a review of literature and conversations about the subject with some members of one of the Fountain Houses in Sweden who had experience of psychiatric inpatient care. Our approach in the study was not to define what a safe or unsafe ward environment could be, nor what situations patients might perceive as violent or threatening, but rather to let the patients define the concepts of safe/unsafe environments and identify situations where these could apply. The main question in the interview guide were about patients’ perceptions of feeling safe or unsafe in the ward (see Additional file 1). The instruction was that the interviewer would let the interview revolve around this issue, but there were some optional open-ended questions and four areas that we could ask about to keep the conversation going. These were on how the patient perceived the importance of (1) the ward’s physical design, (2) the ward’s routines and rules, (3) the staff’s approach to patients and (4) the presence of other patients in relation to feeling safe or unsafe. We also asked if they had encountered situations that they perceived as threatening and/or violent. A normal length of an interview was around 50 min (from 30 min to 1¼ h); 16 interviews were recorded and transcribed verbatim. One patient did not permit recording; notes were taken during this interview.

### Analysis and interpretation

Thematic content analysis with an inductive approach [41, 42] was applied by listening to, and reading, the interviews several times in order to get an overall picture of the material. In the continued reading, we searched places in the text that somehow addressed our research question. Any such place in the text was marked as a meaning unit and moved to a coding sheet. A summary was then made of the meaning unit by giving each unit a code and/or a brief description that was as close to the content as possible. These open codes were then organised under higher order headings. Once this was done, the interpretation work began by creating subthemes/themes with the higher order headings and codes as a basis. The number of preliminary subthemes/themes was gradually reduced. A tentative longer result section, with many quotes, was written in Swedish with relatively many themes and sub-themes. This was used for a discussion with the authors as to which quotes and theme names were adequate and how these would be translated into English. When the final interpretation of the material was done, themes and subthemes were chosen which could give a thick description and best describe the relevant material. The original text was available throughout the analysis and we could go between the whole, and parts of the text. All stages of the process were made by at least two persons; first independently and then discussing and reaching consensus. Important decisions were taken jointly by all five authors. VP is a social worker and Ph.D., TW is a psychiatrist and Ph.D., UH is a registered nurse and Ph.D. student, IN is a psychologist and LK is a social scientist and Ph.D. Our pre-understanding of the subject comes from our professional education, personal experiences of psychiatry and our review of the literature.

## Results

The main results of the analysis with focus on patients’ feelings of safety and unsafety in the ward can be summarized in three themes and nine subthemes (Table 1). The first theme, *predictable and supportive services are necessary for feeling safe,* concerns patients’ experiences with psychiatric services and their perceptions of its ability as a healthcare provider to meet the needs of the patients for treatment and care. The second theme *communication and taking responsibility enhance safety* is about how the participants perceived the daily life at the ward, which was often perceived as being socially poor and boring with non-communicative staff. The third theme, *powerlessness and unpleasant encounters undermine safety* is about how participants addressed problems with encounters that are hard to handle and how they perceived that staff members and co-patients acted in these situations.

**Table 1:** Themes and subthemes constructed in the analysis with focus on patients’ feelings of safety and unsafety at the ward

Theme	Subtheme
Predictable and supportive services are necessary for feeling safe	An unpredictable treatment process A need for structure and routines A desire for a friendly ward climate
Communication and taking responsibility enhance safety	A desire for communicative staff members Asking and waiting Taking responsibility
Powerlessness and unpleasant encounters undermine safety	Keeping away Powerlessness in relation to staff Unpleasant or violent co-patients

### Predictable and supportive services are necessary for feeling safe

Participants described *an unpredictable treatment process* of not knowing whether they would receive adequate medication, other treatment, or care and support. There were descriptions of treatment and care processes that the participants perceived as safe and predictable, but more often the processes were characterized by uncertainty and concern about what would happen after discharge from the ward without appropriate support from psychiatric or social services. Substance users in psychiatric addiction care and other clinics stressed these problems more often than other patients.

I can’t do my social planning in a public toilet. It’s a bit hard to do planning there. My plan is just to get hold of something that will drown my thoughts.

The care described related almost exclusively to medical treatment and waiting for the effect of medication. Several participants described an organization which did not have the capacity to handle the whole patient populations’ needs. They reported of occasions when they themselves or others did not have access to psychiatric care despite experiencing an acute need for care. It was difficult to gain access to treatment, resulting in a delayed treatment, which at times could lead to intoxication by the patient. Once they were admitted to the ward, it was often hard to get an opportunity to meet a doctor or a social worker and it was even harder to get a long-term treatment plan.

The participants expressed *a need for structure and routines* in the wards and often experienced the daily routines, such as fixed time meals and medication, as something positive. The constant presence of staff contributed to safety.

There are always people around you, you don’t have to be alone, alone with your thoughts, and there is nothing to use if you want to hurt yourself either. It feels like there is always someone to talk to. There is a lot of staff in this ward so there is always someone.

Some participants perceived the risk of being exposed to violent behaviour and self-harm as low in the ward in comparison to life on the outside. It felt safe that the door was locked and they had their own contact person and strict routines helping the participants to structure their lives.

Yes, it’s when the doors close and you’re left there standing on your own. It’s really easy to say you’re feeling fine when you’re in here with people around you who’re engaged in your wellbeing.

Closed doors and ward rules could also be perceived as negative if staff members were too rigid or just enforced them to demonstrate their power. One problem, which mainly affected patients in addiction care, was that the mixture of patient groups caused irritation. A mixture of substance users undergoing detoxification and severely mentally ill patients who were disruptive could cause irritation among substance users. This could lead to the patient leaving the ward even though the person felt that they were not ready to leave.

Participants had *a desire for a friendly ward climate* where staff and patients can have a normal social life with a home-like environment, a climate where it was possible to talk and joke with each other and with staff members, but most often there were no social activities around which patients and staff could meet. When activities were promised, there was a relatively high risk that they would be cancelled. In particular, in forensic psychiatry, participants reported that staff did not take into account that the institution was their home. The physical ward environment was also often seen as being problematic since it, together with staff behaviour, constantly reminded patients of being in a closed institution.

They should try to make these kinds of environments as quiet as possible. It’s the same with… doors slamming and things like that. I’m sound-sensitive. I have a bit of hearing impairment so that when there’s any noise it becomes really loud in my head. So I’ve asked the staff not to bang the doors and stuff like that. But the thing is, they’re at work 6–8 h a day. When they go through a door, they just walk through and have these automatic door shutters that bangs the doors closed. They don’t hold the door. You would never behave like that at home…

There were participants with a long experience of psychiatric care who had confidence in the development of psychiatry. They said the staff members are friendlier today than in the 1990s, with fewer abuses and less use of belts, i.e. use of physical restraints.

Because when I came here the ward was more open and brighter with friendly and engaged staff, who were out among us and talking. I can’t really say “us”, but me anyway…

### Communication and taking responsibility enhance safety

Participants had *a desire for communicative staff members* who were available at the ward. Some reported about staff members who did not communicate at all and some could only chat about the weather. Many participants felt that there were only one or two staff members that they had confidence in and who were communicative. A good conversation could mean a lot to the participant, but often they only got very little time to talk undisturbed with their favourite staff member.

Sit down with me so I feel you’re here for me. Don’t go when someone is distressed. It shouldn’t be that when a staff member is sitting and talking to me, someone else comes and disturbs us—some other member of staff just asking a simple question like: “Where’s Anna?”, for example. She shouldn’t disturb our conversation because then I’ll be offended. If I’m in a conversation, a good conversation, it means a lot to me. I can feel a bit better just for the time being.

In particular, night staff were criticized for not communicating with patients. Participants also noticed that communication between professional groups, for example between nurses and doctors, sometimes failed. Staff were also often more interested in their own mobiles and computers than communicating with patients.

Patient: Yes, how can I explain it? It is more the younger ones who come with their phones. They can sit in the dining room, two or three of them sitting next to each other just sitting there with their phones, and I think it’s seriously wrong. They forget the time and forget where they are.

Interviewer: But the older ones that aren’t preoccupied with mobiles what are they doing with their time then?

Patient: In front of the computers: “No, you can wait”…

*Asking and waiting* was a big part of everyday life at the ward. Participants pointed out that the staff did not like to be disturbed by patients asking questions.

When the milk is finished, I shouldn’t have to go into them when they’re sitting and eating at the same time as us and stand in the doorway feeling humiliated and thinking that I’m disturbing them while they’re eating in there. I feel like they’re sitting there, I shouldn’t disturb their lunch… It’s hard to go into them. Disturbing them: “Now the milk is finished.” A potential conflict situation. It just takes a thing like that to trigger a threatening situation. For the feeling I get is: “We don’t have time for you just now when we’re in here. Don’t bother us while we’re eating.” And that sets it off. I have discovered that a lot of conflict arises from this kind of situation.

When patients asked the staff about something they often got the reply: “I’ll get back to you” or they were referred to another staff member. After the patient got the opportunity to ask the question, they often had to wait quite a long time before it was dealt with by staff. Participants noted that many patients were irritated by this slow process and that it could lead to conflicts between staff and patients, upcoming conflicts that staff seldom noticed.

Participants stressed the importance that you, as a patient, are *taking responsibility* for your own rehabilitation. Some participants supported patients who had not reached the same level of rehabilitation as they had done. They sometimes reckoned that they had responsibility towards other patients.

On the other hand, we have a lot of people who go through periods of feeling very bad here… and we bear the responsibility for whether they will live until the next day when they indicate they have suicidal thoughts or have attempted suicide and the likes….. We don’t really know if we’re able to deal with this. Are we aware of what’s going on?…. Safety for me lies somewhere in feeling that I know, in principle, that whatever happens here, we will kind of solve it in a good way. That is some sort of security.

Silent patients risked isolation in the ward since staff seldom talked to them. Sometimes participants took responsibility for talking with these patients. They also commented that patients were responsible for not acting out too badly against the staff.

### Powerlessness and unpleasant encounters undermine safety

So I have no conflicts like that, but there is a lady here who stalks me and gives me drawings all the time. It’s tough but there is no conflict so I choose to keep away.

*Keeping away* was the main strategy that many patients used in order to deal with different problems in the ward. Staff sometimes told them to go to their rooms if there was a risk of violence. Participants described how they did risk analyses of co-patients and staff members. Having your own room was described as being valuable because you could go there when you perceived a co-patient or staff member as unpleasant or aggressive.

I haven’t felt personally that I’ve experienced fear. At the same time, I have wondered: What’s happened now? And how far is he prepared to go? How angry does he get? Is he so angry that he can hit someone? Because it was nearly… yes, you become hesitant.

Participants were aware of the fact that there was a care hierarchy in which the patient was at the bottom. They described *powerlessness in relation to staff* and there were some descriptions of oppressive behaviour from the staff such as the subtle use of power, violations or threats.

“No, we said 9 o’clock.” Yes, but, my partner’s already here and it’s five to nine. Can I not just go out now? “No, at 9 o’clock.” And then they hinder you. I’ve been subjected to that before. Then they delay you, so it ends with the clock being five pasts nine because the nurse doesn’t want to be here when she knew I’d to be out at 9 o’clock.

Participants reported that it was difficult to not to react negatively to staff members with a negative approach. Patients had learnt, particularly in forensic psychiatry, that they have a lot to lose if they showed negative emotions. There were some descriptions of how certain staff refused to “see” patients and also descriptions of stigmatizing behaviour; staff members, for example, using hand sanitizer directly after touching the patient or the patient’s belongings. Participants felt an expectation from the staff that they should not be disturbed too much by patients with questions or worries. They thought that if they did so, there could be negative consequences and, as a result, participants had self-restrictions about talking with the staff. Some female participants expressed that they had been afraid of specific male staff members.

Participants described experiences of *unpleasant or violent co*-*patients*. Violence from co-patients was rare at the ward, but some had witnessed violence between co-patients or between staff and a co-patient. The main problem was that some co-patients were perceived as unpleasant or scary; especially female patients who described scary experiences.

However, there’s a guy who has come in now that goes about like this. He checks that the coast is clear and then talks about horrendous assaults on women all the time.

A female participant from a forensic psychiatric unit was more worried about her male friends than herself, since violence with severe consequences most often occurred between males. Participants from forensic units described more stable wards but, at the same time, they seemed to be more aware than other participants that a serious conflict may occur. A participant had been subjected to a murder attempt in his room by a co-patient after inviting this patient to his room. Staff knew that this patient and another patient were seen as a threat towards him but did not inform the patient about the risk before the incident. Many participants reported, however, that the staff often handled frightened and aggressive patients in a good way. They described how staff often successfully met these patients by acting and talking in a way that made the patient calm. After incidents, staff could talk to patients in order to calm down the situation, but the participants often lacked information about what had happened and whether there was a current or future risk. This lack of information made it difficult for them to assess future risks at the ward; which some of the patients actively did. According to participants, the staff referred to secrecy as an explanation for not giving information to patients.

## Discussion

Participants expressed that they would feel safe if they had a predictable treatment and care process and the ward had a friendly ward climate with supportive routines. They wished they could have communicative staff, access to information they needed and be trusted to take responsibility. In critical situations, they took responsibility off themselves by keeping away from danger when necessary. The importance of the relationship between individual staff members and patients is often emphasized in nursing research. According to participants in this study too, these relations are important, but it would be a mistake to focus solely on the relationship between staff members and patients. Participants emphasised the capacity of the psychiatric organization for giving predictable treatment and care, a good psychical environment and social climate as key factors for feeling safe in the ward.

We did not ask specific questions about the treatment and care process in the interviews but, despite that, the participants talked a lot about these issues. These findings gave the basis for the theme *predictable and supportive services are necessary for feeling safe.* Participants were well aware of the lack of beds in inpatient care and some claimed that this was a reason why they sometimes received delayed care. The lack of access to a safe and effective psychiatric service is a global problem in terms of patient safety [43, 44]. Swedish health care has problems with delayed care, reasons for this may be that different care organisations fail to co-operate around patients’ needs [45] and that Sweden has few beds in comparison to many other OECD countries [46, 47]. In order to create sustainable care processes, patients need to be invited to care planning and given relevant information, thereby enhancing the patients’ feelings of safety. In this study and other studies, patients have reported a lack of information about their treatment, their rights, or the reason why they are in coercive care [26, 28]. They have also reported that they are not often invited to take part in their own treatment and care [22, 25, 26, 30], despite the fact that they would like to be involved and get feedback on the process of recovery [48, 49]. Participants were aware of organizational problems in psychiatric outpatient care and social services and as a result of these problems, they suspected that after discharge, they would not get the treatment, care and support necessary in order to recover. Another problem described was that the participant or other patients left the ward prematurely if they did not feel capable of handling the situation regarding problematic co-patients. This is consistent with previous research; the mixture of patients at a ward can be a source of triggers for violence and absconding [11, 24].

This study confirms earlier studies that socially poor and boring wards with non-communicative staff create distress [11, 50] and are, according to participants, something that could trigger aggression from patients, while *communication and taking responsibility enhance safety.* Access to communicating staff members with whom patients can talk about their experiences can be a way of making the patients situation understandable and promote the feeling of safety [49]. In another study with staff members in three of the participating clinics in this study, staff emphasized the importance of establishing a relationship with patients, talking to them and indicated that knowing each other had a preventing effect on violence [40]. Despite this awareness of the importance of communication, the participants in this study did not perceive the staff as communicative in general, nor providing them with information. Participants were assessing risks at the ward regarding co-patients and staff members. Just like another study [39], they considered this to be difficult since they did not receive information about risks from the staff.

Participants described that *powerlessness and unpleasant encounters undermine safety.* Some participants with a long experience of psychiatric care were positive about the development of psychiatry. They perceived less abuse of patients and felt more respected by staff nowadays than a few decades ago. Participants were impressed by the ability of some staff members to interact with aggressive or frightened patients in a calm manner and using de-escalation methods. Some positive results in this study, in contrast to some other studies, was that no one reported problems with theft or problems with co-patients using drugs or alcohol [38], nor did they feel that patient safety was dependent on the presence of male staff [39]. Research confirms what a female participant said in the result section, namely, that men are more likely to be involved in violent incidents with more severe consequences than women, although a female-only ward may have at least as many incidents of violence and self-harm as a mixed ward [51]. At the same time, several participants reported inappropriate behaviour by other staff members, such as aggressive, stigmatizing or oppressive behaviour. This is in contrast to Stenhouse’s study [39], where patients only reported problems with staff who did not engage in patient work, but not about inappropriate behaviour. Some stories in our study give the impression that there are staff members who seem to try to provoke violent behaviour from patients. In most of these cases, the patients understood that it was no idea to protest since it could end with coercive measures, which were also reported as occurring. Some participants were exposed to stigmatizing behaviour by staff members, which is serious since feelings of being stigmatized might delay recovery and is also correlated with suicide [52]. If staff members in our study did communicate risks with patients concerning violence, it was only after violent incidents, and then the purpose was to calm down the patient emotionally rather than giving facts about the situation or security measures.

Staff behaviour can trigger or de-escalate violence in the ward, so it is important to create organizational values which can strongly counteract provocative, oppressive and stigmatizing behaviour toward patients and start to combat stigma [25]. The management can give support to the staff team by providing the conditions for a good functioning ward with a clear purpose [11, 53]. This should include staffing levels that are sufficient for safety, improving staff responses to violence and giving them opportunities to spend time with patients [53]. With organizational support and a supportive ward manager, staff teams would find it easier to modify their anxiety and frustration which would promote staff-patient interaction and safety [11, 53]. These kind of interventions could give the fundament for cohesive staff teams that are more content with their psychosocial climate at work, are morally committed, and show more understanding and positive appreciation towards patients, thereby also creating a ward climate that can have a positive primary prevention effect on violence [10, 53].

This study had a variety of different kind of wards with different specialities, different treatment, different patient groups, different staff groups and differences in management styles and organizational values. Our aim was to describe feelings of being safe in psychiatric inpatient care in general. Since we only had one clinic with few patients from each specialty, we cannot draw conclusions about differences between specialties. A weakness of the study was that we had no control over the inclusion process of participants or the exact number of patients that refused to participate. It was the managers who decided when a patient was sufficiently healthy to be interviewed. We did not collect extensive information about the patients because the act of just writing their name on the consent form felt difficult for some patients. We managed to get a spread in age, but not an even gender distribution. Instead, the study contributes to our insight into how patients perceive feelings of being safe or unsafe during their stay in a psychiatric ward and can give some ideas about what we should improve to achieve effective primary prevention.

To sum up, patients in this study were worried about the unpredictable treatment and care process, a boring social climate in wards, and a lack of communication with staff. All these factors are important from the primary violence prevention and recovery perspective. There are programs such as Safewards [11] and Star Wards [20] that have several elements that participants in this and other studies have requested. These elements include creating a socially friendly climate, starting treatment in form of talking therapies and self-care programs immediately, involving patients in their care planning and giving them the opportunity to talk with a staff member every day. A development in line with this could make patients feel more safe and secure since many wards implementing these programs have experienced changes, with less patient aggression than earlier [20, 54].

Many patients in this study were afraid of discharge and wondered whether they would receive adequate treatment and care. For substance users, in particular, but probably also for other groups, it may be necessary to have services that are more assertive, with improved coordination of health and social services in order to have the capacity to secure treatment and care processes [55, 56].

Participants in this study tried to assess the future risk of violent incidents. It is, therefore, important that staff provide patients with general information about the safety situation and what measures the staff team can take. This kind of safety issue can develop into an ethical dilemma between respecting an individual patient’s right to confidentiality and other patients’ need of information about safety. Despite some very positive results with a number of patients describing communicative staff members and the use of de-escalation methods, there were also far too many descriptions of staff with an aggressive approach, indicating that some units have organizational values that might inadvertently lead to some staff members behaving aggressively or stigmatizing patients. It seems there is a need for ongoing work, not only with implementation of de-escalation techniques but also with organizational values [25, 53, 57].

## Conclusions

Our results indicate that creating reliable treatment and care processes, a stimulating social climate in wards, and better staff-patient communication could enhance patient perceptions of feeling safe. It seems to be important that staff provide patients with general information about the safety situation at the ward, without violating individual patients right to confidentiality, and to have an ongoing process that aims to create organizational values promoting safe environments for patients and staff.

## Additional file

**Additional file 1.** The main question in the interview guide were about patients’ perceptions of feeling safe or unsafe in the ward.
