# Supplementary material for: Feeling safe or unsafe in psychiatric inpatient care, a hospital-based qualitative interview study with inpatients in Sweden
Source: Int J Ment Health Syst. 2019 Apr 8;13:23. doi: 10.1186/s13033-019-0282-y (PMC6452515; doi:10.1186/s13033-019-0282-y)
Supplement: Supplementary file 1 — Additional file 1. The main question in the interview guide were about patients’ perceptions of feeling safe or unsafe in the ward. [file 13033_2019_282_MOESM1_ESM.docx]

**Additional file:** Feeling safe or unsafe in psychiatric inpatient care, a hospital-based qualitative interview study with inpatients in Sweden.

# Instructions to interviewer

The instruction to interviewer was to focus on the main question and to interject questions from below if, and when, appropriate during the patient’s narrative.

Use prompting and be like a "co-researcher", i.e. that interviewers and participants focus on the subject and aim to find out about the participant’s experiences and thoughts about their situation.

- Would you like to develop it a little bit more?
- Tell me more about…!
- Can you tell me what you mean by… (“words or concepts”)!
- I wonder if I got this right ... you mean that ...
- What’s the difference between .… and …. (You say “they don't care” or “they care”: What’s the difference?)
- I would like to understand better what you’re thinking about when you said …… about…

# The interview guide

## Introduction

The interviewer…

- introduces him/herself.
- briefly explains the purpose of the study and presents, in short, the topic for the interview with an emphasis on violence and feelings of being safe or unsafe.
- gives an assurance of confidentiality.
- explains how the material will be handled - recording, transcribing, reporting, confidentiality.
- gives the opportunity for the participant to ask questions about the study.
- asks the participant to write the consent form.
- asks the participant to briefly present him-/herself: saying a few short words about his/her own background and relationship to ward environment.

## Main question

I would like to know about your experiences and feelings of the ward as a safe place and when you find the ward an unsafe place to be in.

- If safe, please describe it in more detail

- If not safe, please describe it more closely

## Additional questions

I wonder if there have been any conflicts between you and nurses, doctors, nursing staff and other patients during your stay at the ward. I mean a conflict situation where you suddenly felt insecure or experienced the environment as threatening.

- Why do you think this incident happened?
- What happened during the incident?
- How was your relationship with the staff and other patients during the conflict?
- How did the staff respond during it all?
- How communicative were the staff while it was happening?
- How communicative were you during?
- How did it end?
- What happened after that? How did the staff treat you and others after the incident? (Did they give information to you?)
- How do you view their part in it? Good, bad ... please tell me more about it.
- Did this have any consequences for you or others (e.g. where things got worse or privileges were withdrawn?)
- How do you think you will react if a similar incident happens in future in the ward?
- If you think that staff behaved badly, ... what would you like the staff to do instead?
- What kind of feelings do you have about the incident when you look back at it?
- Suggestions for improvement?
- Why do you think they treated you that way?
- Do you feel that people who do not know you treat you in similar way as staff in the ward? If yes, why do you think it is so?
- What’s your relationship with the staff like?
- How do you find the relationship when there is conflict between you and them?
- What’s the atmosphere like if you get angry for some reason?
- What are nurses, doctors and caregivers like if you are dissatisfied with something in the ward?
- How would you describe a good staff member, i.e. doctors, nurses, caregivers? How would you describe a bad staff member?
- Does it feel like you are getting help and support for your difficulties here? Do you find it worthwhile being here in the ward?
- How do you think your experience differs from other patients?
